# Assessing individual patients’ knowledge of benign versus malignant skin lesions in the dermatology clinic population

**DOI:** 10.1097/JW9.0000000000000032

**Published:** 2022-07-25

**Authors:** Kristin Lee, Ngoc Nguyen, Meghan Fuzzell, Eleanor Tung-Hahn, Jeave Reserva, Neelam Balasubramanian, Rebecca Tung, Murad Alam, Thomas Stasko

**Affiliations:** a Division of Dermatology, Loyola University Chicago, Maywood, Illinois; b Department of Dermatology, University of Oklahoma Health Sciences Center, Oklahoma City, Oklahoma; c University of Oklahoma College of Medicine, Oklahoma City, Oklahoma; d Loyola University Chicago Stritch School of Medicine, Maywood, Illinois; e Departments of Dermatology, Otolaryngology and Surgery, Northwestern University Feinberg School of Medicine, Chicago, Illinois

**Keywords:** nonmelanoma skin cancer, melanoma, Skin cancer education, patient education

## Abstract

Skin cancer education targeted to patients’ needs is a goal of practicing dermatologists. Data regarding dermatology patients’ baseline knowledge regarding skin cancer could aid clinicians in tailoring education efforts.

**Objective:**

To help quantify existing patients’ existing visual recognition of skin cancer and common benign lesions, with the goal of helping to provide more targeted and meaningful education to patients.

**Methods:**

Two hundred forty-four adult patients from the dermatology clinics at University of Oklahoma and Loyola University Chicago were surveyed using digital images and questions regarding personal and family history of skin cancer, sun protection practices and sun protection knowledge.

**Results:**

Of the 244 subjects, 43% percent had a positive personal history of skin cancer, 40% had a positive family history. Scores differed minimally by personal history of skin cancer (*p* = .37) but differed more markedly by family history of skin cancer (*p* = .02).

**Limitations:**

Lack of generalizability to the general public, age range of subjects.

**Conclusions:**

There are knowledge gaps within the dermatology patient population regarding common benign and malignant skin lesions.

**What is known about this subject in regard to women and their families**

- Skin cancer is the most common malignancy in the United States
- Skin cancer is related to UV light exposure, which is relevant for women and their families who have outdoor exposure

**What is new from this article as messages for women and their families?**

- Knowledge regarding skin cancer and sun- safe practices varied between subjects
- Subjects with a positive family history of skin cancer scored higher on the photo survey compared with those without a family history of skin cancer

## Introduction

The lifetime risk of developing skin cancer in the United States is approximately 1 in 5,^1^ and the incidence continues to rise.^2–6^ Nonmelanoma skin cancer (NMSC) makes up the vast majority of cases, causing significant morbidity, but low case- fatality. Melanoma accounts for a much smaller proportion, yet it is the cause of 65% of skin cancer-related deaths.^7–9^ Mortality from malignant melanoma continues to increase among men and women over the age of 65 but appears to be stabilizing in the younger population.^10–13^ Treatment of skin cancer poses a significant economic burden in the United States with an annual average cost of $8.1 billion.^14^ Fortunately, early detection and treatment of most skin cancers results in an overall 5-year survival rate of 95%.^13^ A study by Berwick et al. suggested that performing skin self-examinations could significantly reduce melanoma incidence and mortality.^15^

It is well established that UV radiation exposure plays a major role in the development of skin cancer.^16–18^ Certain risk factors make an individual more susceptible to its harmful effects include light skin and eye color, red or blonde hair, and a tendency to freckle.^19–22^ A prior history of melanoma or NMSC substantially increases the risk of developing a subsequent skin cancer.^23–25^ Unfortunately, several studies have shown that while a previous history of skin cancer resulted in increased sun protective practices compared with controls, many patients continued to engage in unprotected episodes of sun exposure resulting in a sunburn prevalence similar to controls.^26–28^ Low levels of perceived skin cancer risk, inconvenience, and lack of knowledge on skin cancer and sun protection strategies are possible explanations for this paradox.^29–33^

Given that the most common modifiable risk factor (UV exposure) is preventable and early detection and treatment can significantly reduce the associated morbidity, mortality, and economic consequences, effective education to improve public knowledge of skin cancer and sun protective behaviors would be tremendously beneficial. Education targeted to patient’s needs would be optimal; however, such targeting is difficult without knowing patients’ levels of knowledge regarding skin cancer. If skin self-examinations and self-detection of skin cancer are to significantly improve prognosis, it would be helpful to determine patients’ abilities to recognize worrisome versus benign skin lesions. In this study we sought to determine baseline knowledge of benign and malignant skin lesions in the general dermatology clinic patient population by their ability to visually differentiate between cancerous versus noncancerous skin lesions. A few similar studies have been carried out in Australia,^34–36^ such as Baade et al.^35^ who found that when comparing general practitioners to community members, the probability that the general practitioners thought a given photo of a pigmented lesion was malignant was significantly higher than that of the community members. However, to our knowledge, no survey-based studies utilizing photographs of skin lesions have been done in the patient population in the United States.

## Methods

**Fig. 1.**
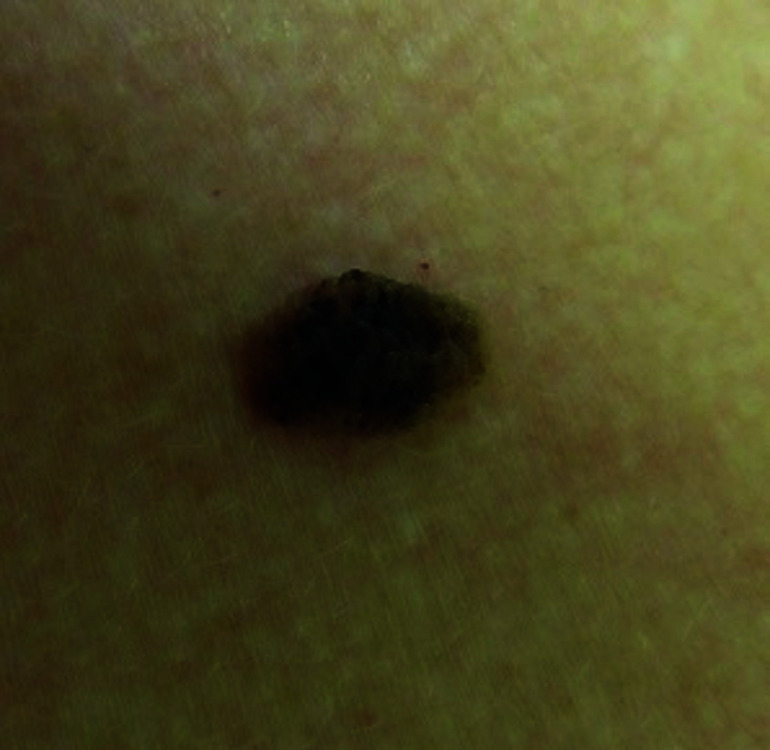
Seborrheic keratosis

**Fig. 2.**
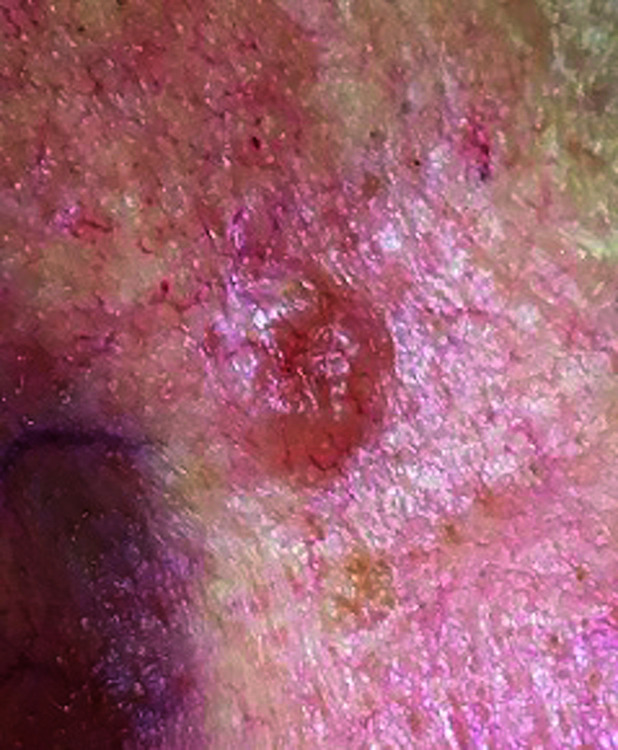
Basal cell carcinoma

**Fig. 3.**
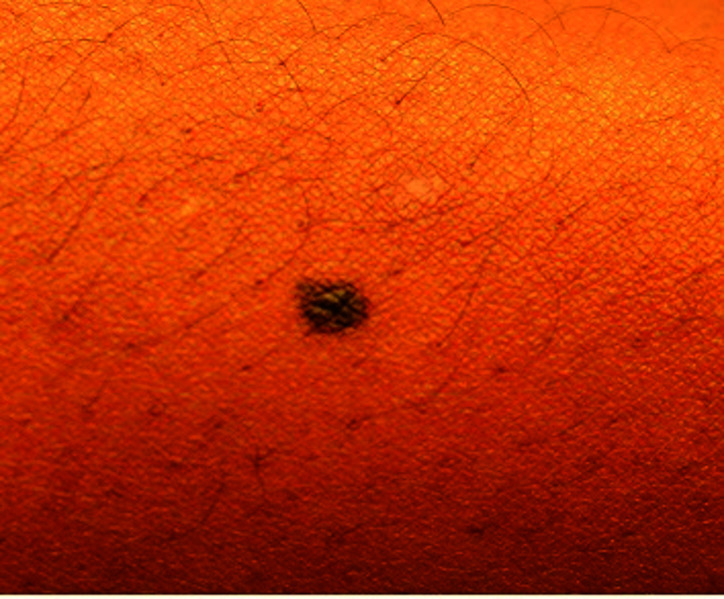
Benign nevus

**Fig. 4.**
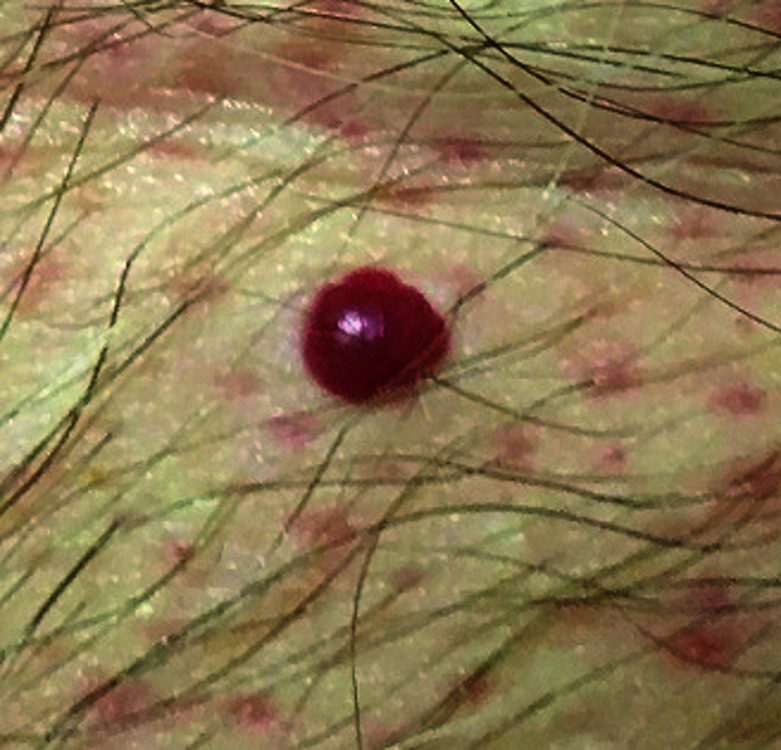
Cherry angioma

**Fig. 5.**
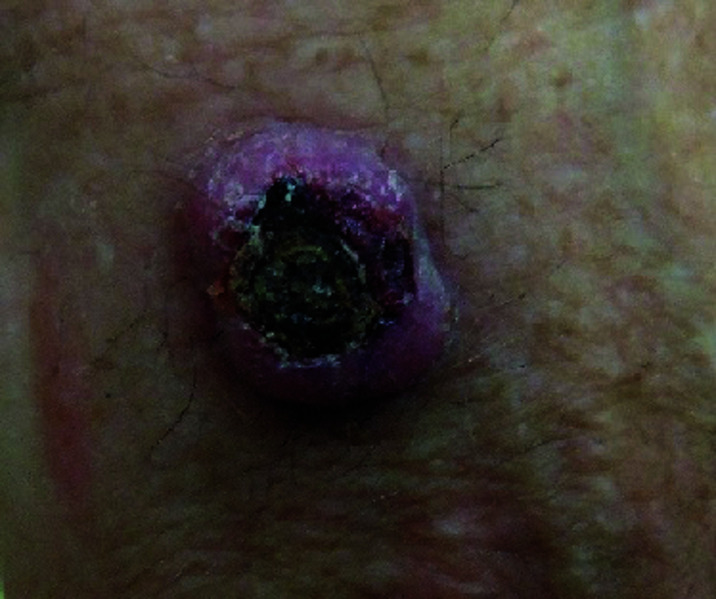
Squamous cell carcinoma

**Fig. 6.**
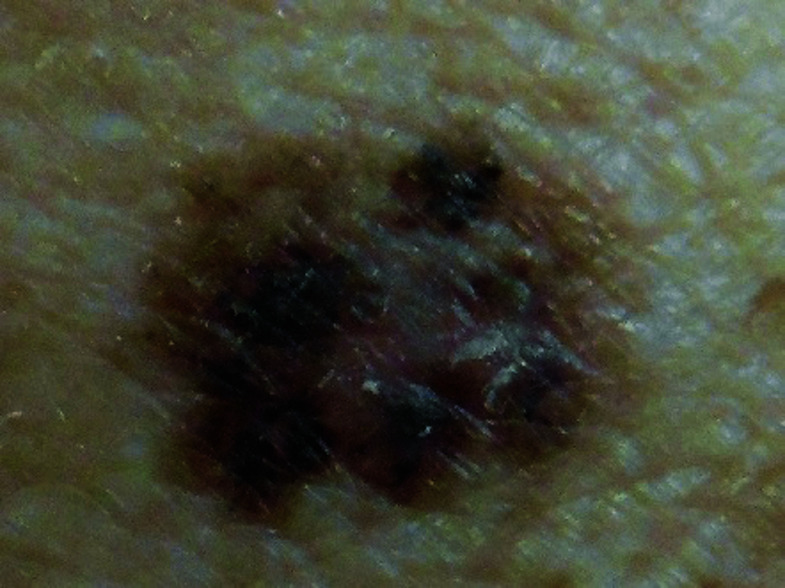
Melanoma

**Fig. 7.**
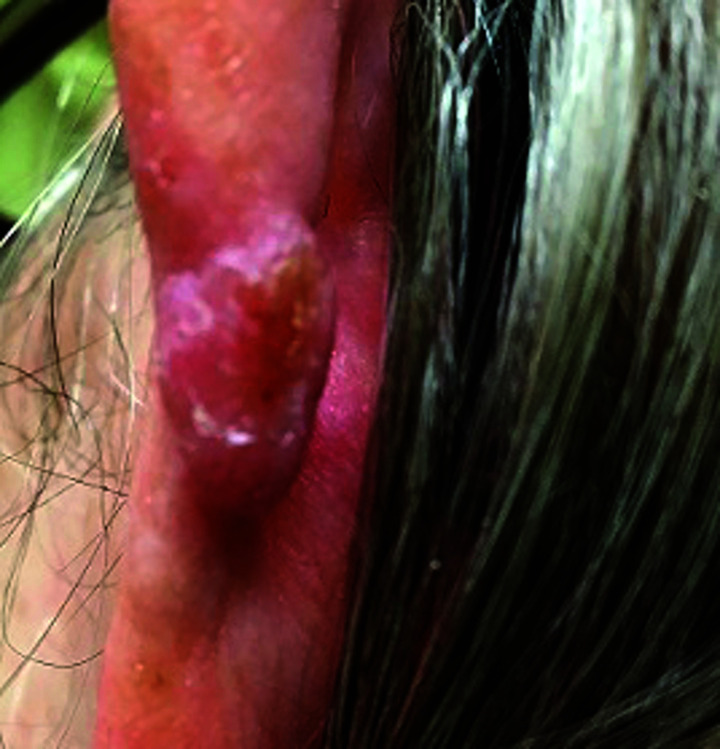
Basal cell carcinoma

**Fig. 8.**
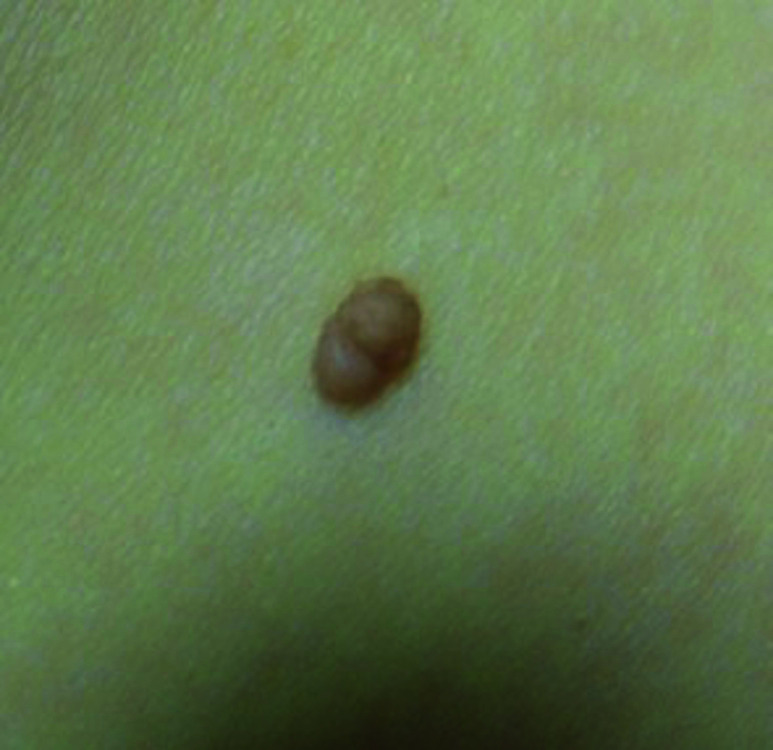
Benign nevus

**Fig. 9.**
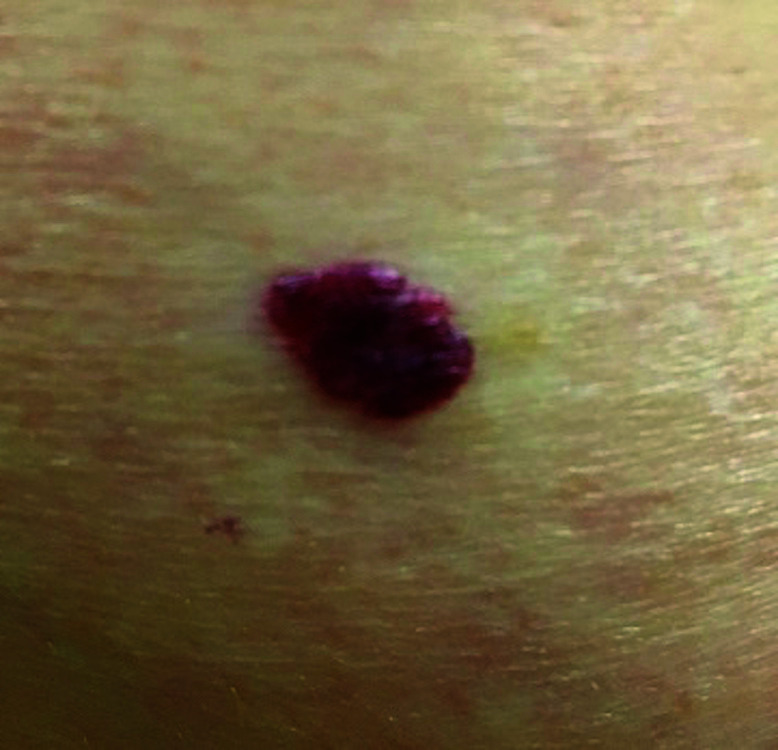
Cherry angioma

**Fig. 10.**
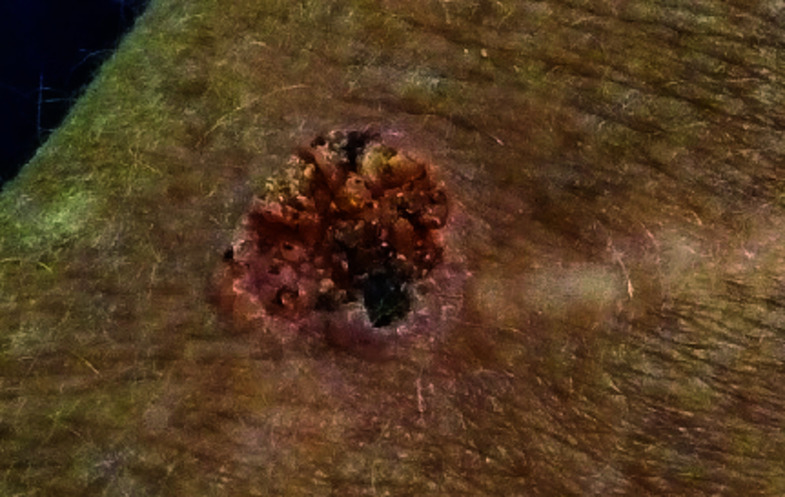
Squamous cell carcinoma

**Fig. 11.**
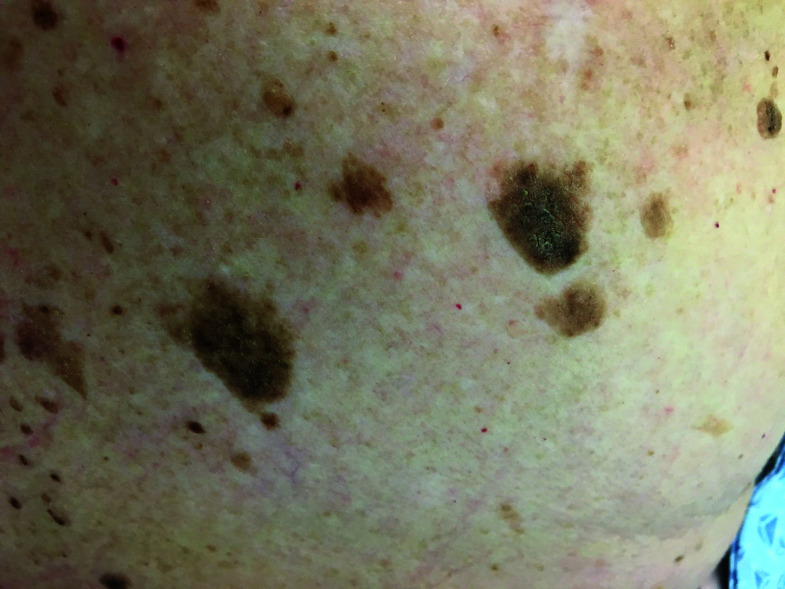
Seborrheic keratosis

**Fig. 12.**
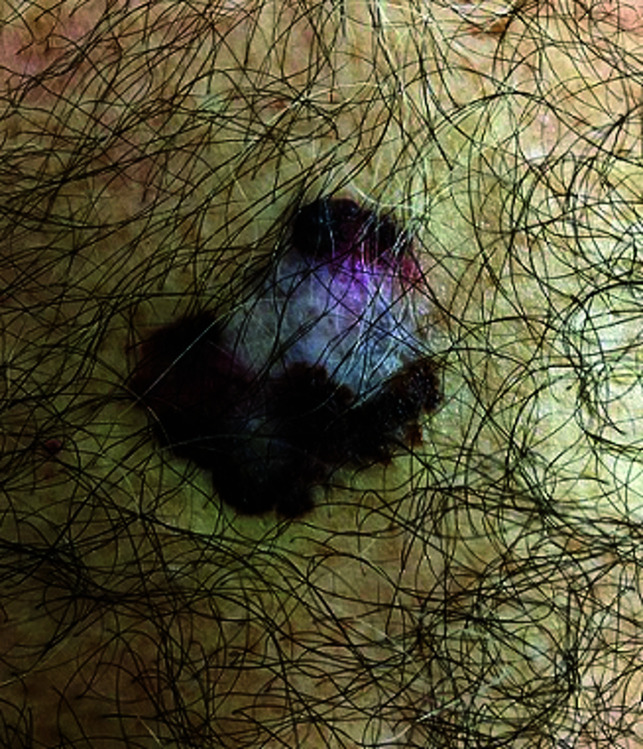
Melanoma

**Fig. 13.**
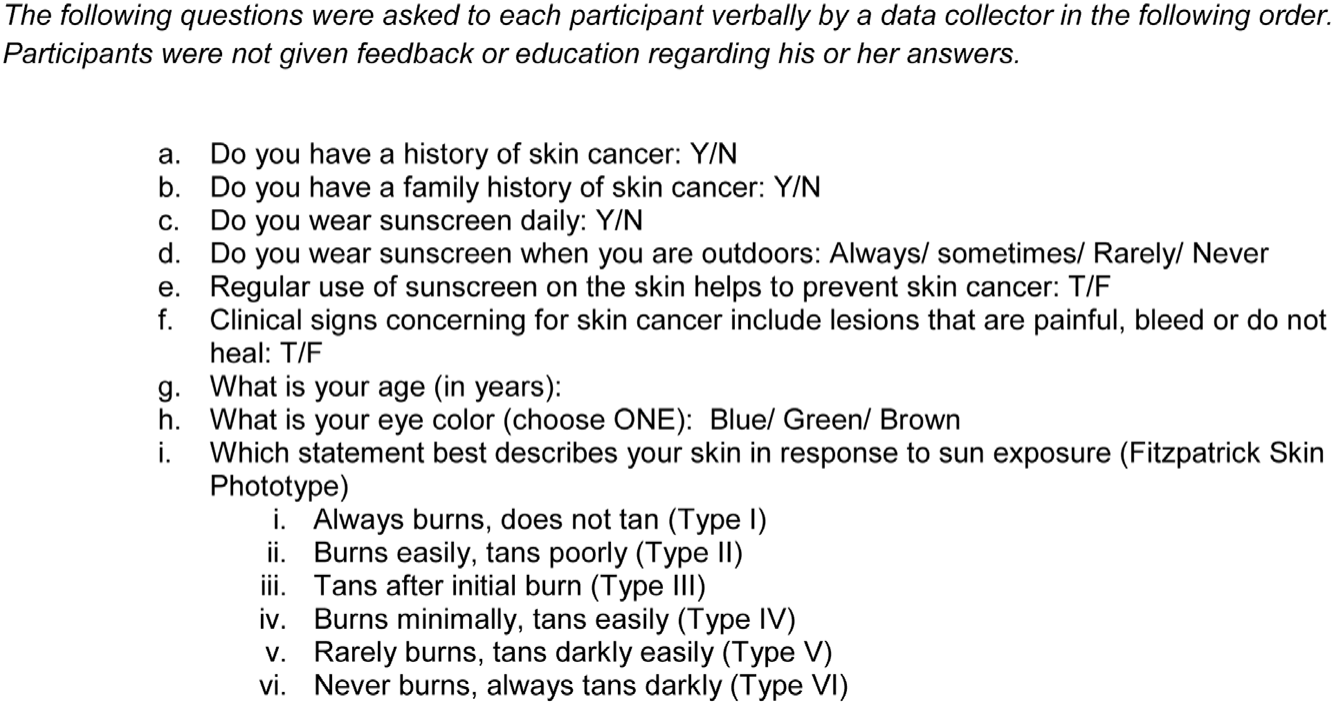
Participant questionnaire: the following questions were asked to each participant verbally by a data collector in the following order. Participants were not given feedback or education regarding his or her answers.

A total of 244 participants were included in the study. One hundred consecutive patients from the outpatient dermatology clinic at the University of Oklahoma and 144 consecutive patients at Loyola University Chicago dermatology clinic were surveyed. Approval for this study was obtained from each site’s respective Institutional Review Board (IRB). Adult patients ages 40-90 seen in the dermatology clinic were asked at the end of their clinic visit if they were interested in participating in the survey. Written informed consent was obtained and all surveys were conducted in a private room to maintain confidentiality. Each participant was presented with 12 digital images of benign and malignant skin lesions and were asked to identify each image as “cancer” or “not cancer” (see Figs. 1-12). Participants were not asked to make diagnoses. Participants’ scores were calculated as the sum of correct responses to the photographs. The photographs used in this study were reviewed and deemed acceptable representations of their diagnoses by four board- certified dermatologists who were not involved in the study. The surveyor then asked each participant a series of questions covering the participant’s family and personal history of skin cancer, skin cancer knowledge, sun protection practices, and Fitzpatrick skin type (Fig. 13). In addition, a retrospective chart review was conducted for each participant to determine the presence and number of biopsy-confirmed skin cancers. Participants were not provided any post-survey education as part of this study. Linear mixed models with random intercepts for the institution were used to determine difference in score by each patient characteristic. All analyses were performed using SAS Version 9.4 (Cary, NJ).

## Results

Of the 244 participants who completed the survey, 57% were female and 43% were male. The mean age ± SD was 61 ± 15. Forty percent of participants reported a family history of skin cancer, and 43% had a personal history of biopsy- confirmed skin cancer which was ascertained through chart review (Table 1). We found an inverse association between participant age and ability to discern lesions correctly on the photo survey. With each year that the participant’s age increased, the average score decreased by 0.02 points (*p* = .01). Participants with a family history of skin cancer had a significantly higher mean score than those without a family history of skin cancer (*p* = .02) while a personal history of skin cancer was not associated with a higher score (*p* = .47). There was no significant difference in mean score between males and females (Table 2). The majority of participants were able to correctly identify malignant lesions while among benign lesions, seborrheic keratoses and cherry angiomas were most often incorrectly identified as being malignant (Table 3). Ninety percent of participants recognized the clinical signs concerning for skin cancer, and 95% agreed that regular use of sunscreen helps prevent skin cancer. Only 28% of participants reported daily use of sunscreen. When asked specifically about the frequency of sunscreen use when outdoors, participants responded: “Always” 23%, “Sometimes” 39%, “Rarely” 21%, and “Never” 16%. There was no difference in score between Fitzpatrick skin type (Table 2).

**Table 1:** Participant responses to questionnaire (see Fig. 4)

History of skin cancer	n (%)
No	139 (57)
Yes	105 (43)
Family history of skin cancer	
No	146 (60)
Yes	98 (40)
Daily sunscreen use	
No	175 (72)
Yes	69 (28)
Sunscreen use frequency	
Always	57 (23)
Sometimes	96 (39)
Rarely	52 (21)
Never	39 (16)
Regular use of sunscreen helps prevent cancer	
False	12 (4.9)
True	232 (95)
Clinical signs concerning for skin cancer question	
False	25 (10)
True	219 (90)
Fitzpatrick skin type	
I	19 (7.8)
II	39 (16)
III	83 (34)
IV	55 (23)
V	34 (14)
VI	14 (5.7)

**Table 2:** Effects of patient characteristics on score

	Beta (SE)	*p*
Age	–0.02 (0.01)	.01
	mean score (SE)	
Sex		.58
Female	8.03 (0.18)	
Male	7.89 (0.19)	
History of skin cancer		.47
No	8.04 (0.17)	
Yes	7.81 (0.22)	
Family history of skin cancer		.02
No	7.80 (0.18)	
Yes	8.26 (0.20)	
Daily sunscreen use		.79
No	7.97 (0.17)	
Yes	8.02(0.22)	
Fitzpatrick skin type		.86
I	8.04 (0.39)	
II	8.14 (0.27)	
III	7.95 (0.21)	
IV	8.10 (0.24)	
V	7.71 (0.29)	
VI	7.79 (0.43)	
History of biopsy proven skin cancer		.37
No	8.05 (0.17)	
Yes	7.86 (0.20)	

N = 244. *p* values calculated using separate linear mixed models with random intercepts for the site.

SE, standard error.

**Table 3:** Image survey answers by question

	Patient response to images		
	Malignant	Benign	
	%	%	% Correct
**No. Answer key**	74	26	26
1. Seborrheic keratosis			
2. Basal cell CA	50	50	50
3. Benign nevus	52	48	48
4. Cherry angioma	32	68	68
5. Squamous cell CA	91	9	91
6. Melanoma	93	7	93
7. Basal cell CA	59	41	59
8. Benign nevus	18	82	82
9. Cherry angioma	69	31	31
10. Squamous cell CA	86	14	86
11. Seborrheic keratosis	31	69	69
12. Melanoma	93	7	93

N = 244.

## Discussion

The findings in this study demonstrate that there is a gap in knowledge within the dermatology clinic patient population regarding the recognition of benign and malignant skin lesions. While malignant melanoma and squamous cell carcinoma were most often identified correctly as malignant, basal cell carcinoma was not as commonly identified correctly. Benign melanocytic nevus was identified as benign by a majority of participants but cherry angioma and seborrheic keratosis were more commonly identified incorrectly as malignant. A possible explanation for this could be that patients are mainly educated on the “ABCDE’s”(asymmetry, border, color, diameter, evolution) of melanoma leading them to identify any lesions with such characteristics as malignant. Since the images of basal cell carcinomas included in the photo survey lacked those characteristics, participants were more likely to identify them as benign. This could also explain why seborrheic keratosis was often identified as malignant due to its dark pigmentation. Of the two cherry angioma images included in the survey, image 4 was more round with regular borders whereas image 9 appeared to be more raised with irregular borders which likely explains why a majority (68%) correctly identified image 4 as benign compared with 31% for image 9. This demonstrates that patients are at least familiar with some of the signs of skin cancer. This is further supported by the observation that 90% of participants correctly answered “true” for the clinical signs concerning for skin cancer (“*Clinical signs concerning for skin cancer include lesions that are painful, bleed or do not heal: True or False?”,* see Fig. 1).

Participants were asked if they had a personal or family history of skin cancer. Participants who had a positive family history of skin cancer scored significantly higher on the photo survey than those without a family history, but surprisingly, there was no significant difference in score between participants with and without a personal history of skin cancer. It may seem logical that if a patient had previously been diagnosed and treated for a skin cancer, he or she would be more knowledgeable about recognizing its appearance. Interestingly, this was not the case based on our data. This is a concerning finding, given that people who have a history of prior melanoma or nonmelanoma skin cancer are at substantially increased risk of developing another skin cancer.^24–25,37–39^ Of note, increasing subject age was correlated with lower knowledge scores. These points support the suggestion that when possible, dermatologists should take the time to review the characteristics of suspicious lesions with patients so that patients may know when to seek medical attention for a new or changing lesion.

Participants were also asked a true or false question on whether regular sunscreen use helps prevent skin cancer in which 95% answered correctly. Despite this, only 28% of participants reported actually using sunscreen on a daily basis. Further, only 62% of participants used sunscreen when outdoors “Always” or “Sometimes” while 37% “Rarely” or “Never” did. These numbers are similar to previous studies examining sunscreen usage in the general population. In one study, researchers reported that 42% of respondents rarely or never used sunscreen^40^ while another found that 26% use it most or all the time,^41^ consistent with the 23% that “Always” used sunscreen in our study. It is clear that there is a difference between awareness of the effectiveness and utility of sunscreen usage in preventing skin cancer and the daily, practical habit of sunscreen application.

The limitations of this study include the lack of generalizability to the general public, as the study group was confined to general dermatology clinic patients. Thus, the results may be skewed since individuals who regularly see a dermatologist may have received more skin cancer education than those who do not. However, if true, this would support the authors’ position that increased education results in decreased gaps in knowledge, resulting in earlier skin cancer diagnosis and treatment. The age range of subjects also does not extend to young adults and teens, so we cannot extrapolate younger persons’ knowledge on these topics. In addition, the authors acknowledge that, while over 75% of study participants were rated Fitzpatrick skin types III or higher, the majority of the photographs used in the study depicted skin lesions on Fitzpatrick skin types I–III, which may have affected participants’ scores, especially those participants with higher Fitzpatrick skin types.

In conclusion, while patients may be familiar with some of the signs and symptoms of skin cancer, it is important for dermatologists to educate patients that not all skin cancer exhibit characteristics that conform to “ABCDE” or other checklist features. This is particularly true of basal cell carcinoma. Increased focus should also be placed on frequent education of patients with a personal history of skin cancer regarding skin cancer awareness, recognition of signs and symptoms of cutaneous malignancy and sun protection. Lastly, patients should continue to be encouraged to use sunscreen regularly while outdoors.

## Author contributions

The opportunity to share an accurate and detailed description of their diverse contributions to the published work. The following are the types contribution for your reference: Conceived and designed the analysis; Collected the data; Contributed data; Performed the analysis; Wrote the paper.

## Conflicts of interest

None.

## Funding

None.

## Study approval

The author(s) confirm that any aspect of the work covered in this article that has involved human patients has been conducted with the ethical approval of all relevant bodies.
